# IgG4-Related Disease in a Urachal Tumor

**DOI:** 10.1155/2014/275850

**Published:** 2014-08-18

**Authors:** Travis W. Dum, Da Zhang, Eugene K. Lee

**Affiliations:** ^1^Department of Urology, University of Kansas Medical Center, 3901 Rainbow Boulevard, MS 3016, Kansas City, KS 66160, USA; ^2^Department of Pathology, University of Kansas Medical Center, 3901 Rainbow Boulevard, Kansas City, KS 66160, USA

## Abstract

IgG4-related disease is a newly recognized fibroinflammatory disorder that has the ability to affect nearly every organ system. It is characterized by tumefactive lesions and fibrosis and closely mimics neoplasms. Only one case of IgG4-related bladder mass has been reported in the literature, but there are no reports of IgG4-related disease in a urachal mass. Herein, we report a 26-year-old male who initially presented with symptoms of recurrent UTI. Work-up revealed a 6 cm urachal tumor, a 1.4 cm pulmonary lesion, and mediastinal lymphadenopathy; all metabolically active on PET scan and suspicious for urachal adenocarcinoma. Lung lesion fine needle aspiration and TURBT pathology revealed inflammation but no evidence of malignancy. The patient underwent a partial cystectomy and umbilectomy with pathology demonstrating dense plasmacytic cells, a high rate of immunohistochemistry staining positive for IgG4 plasma cells, a storiform pattern of fibrosis, and an obliterative phlebitis. Furthermore, the patient had an elevated serum IgG4 level of 227 mg/dL (range 2.4–121 mg/dL). IgG4-related disease is a newly recognized fibroinflammatory disorder that can mimic neoplastic processes and a high index of suspicion and accurate tissue pathology is necessary for an accurate diagnosis.

## 1. Introduction

IgG4-related disease is a newly recognized condition characterized by a fibroinflammatory process that leads to lymphoplasmacytic infiltration containing IgG4-positive plasma cells, fibrosis, and tumefactive lesions than can be locally destructive [1]. Elevated serum IgG4 in a group of patients with sclerosing pancreatitis was first reported in 2001, a disease now known as autoimmune pancreatitis [2]. Later, recognition of systemic involvement of fibroinflammatory lesions involving IgG4 led to development of the term IgG4-related disease and has since been described in nearly every organ system [1, 3].

IgG4-related disease can involve the genitourinary system. Reported cases include interstitial nephritis, ureteral obstruction due to ureteral pseudotumor or retroperitoneal fibrosis, prostatitis, and epididymoorchitis [4–7]. There has only been one reported case of bladder involvement by IgG4-related pseudotumor in an elderly female [8]. Herein, we report a 26-year-old male who was found to have an IgG4-related urachal tumor.

## 2. Case Presentation

**Figure 1:**
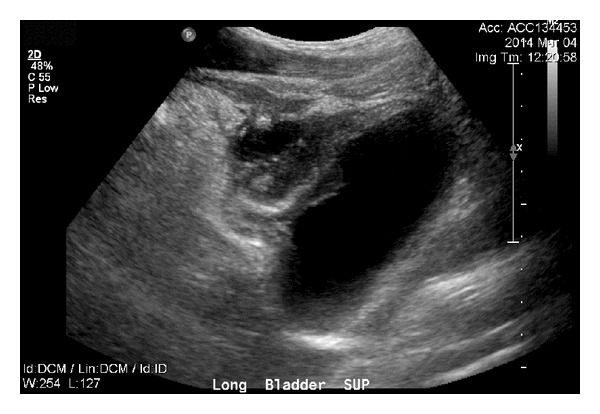
Ultrasound image of 6 cm urachal mass.

**Figure 2:**
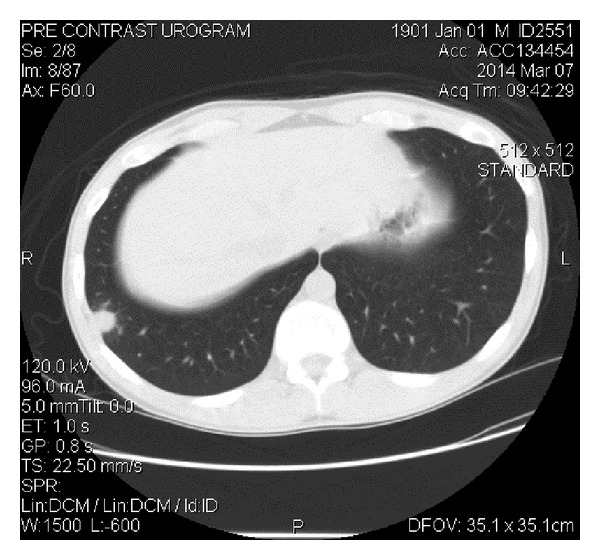
CT chest demonstrates right lung base nodule.

**Figure 3:**
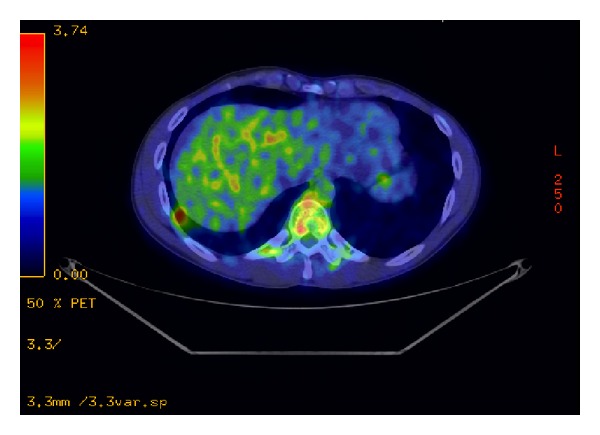
PET CT scan shows hypermetabolism of lung base nodule.

**Figure 4:**
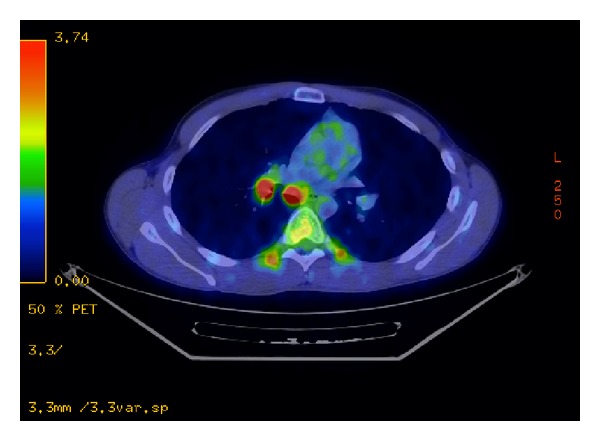
PET CT demonstrates hypermetabolic mediastinal lymph nodes.

**Figure 5:**
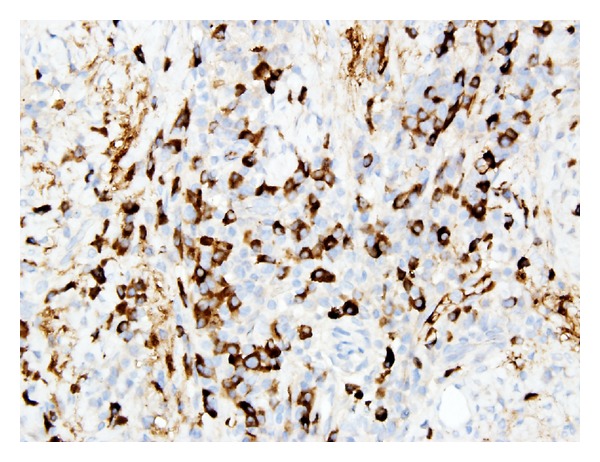
IHC for IgG4+ cells.

**Figure 6:**
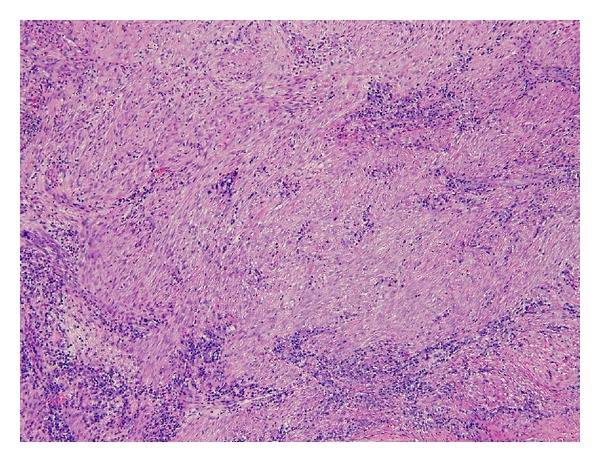
Storiform pattern of fibrosis.

**Figure 7:**
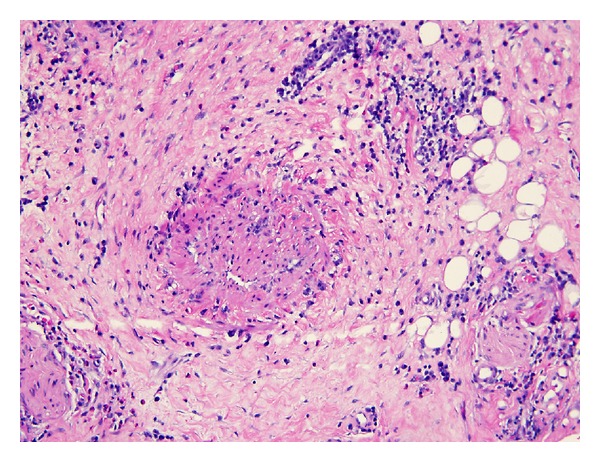
Obliterative phlebitis.

The patient is a 26-year-old male who presented with symptoms of recurrent urinary tract infections despite antibiotic therapy. An abdominal ultrasound revealed a 6-cm mass superior to the bladder consistent with a urachal tumor (Figure 1). Computed tomography (CT) of the chest identified a 1.4 cm right lung lesion suspicious for metastatic disease (Figure 2). 18-F FDG-PET scan was performed and demonstrated increased metabolic activity of the urachal mass, the lung lesion (Figure 3), and two enlarged mediastinal lymph nodes (Figure 4). Cystoscopy revealed a tumor at the dome of the bladder and transurethral resection of the bladder tumor revealed chronic inflammation and fibrosis. Fine needle aspiration of the lung mass demonstrated inflammation but no evidence of malignancy. A partial cystectomy with umbilectomy was performed. The pathology demonstrated no evidence of malignancy but a high density of lymphoplasmacytic infiltration with 3–90 IgG4-positive plasma cells per high-powered field (Figure 5). Furthermore, the patient's pathology demonstrated a storiform pattern of fibrosis (Figure 6) and an obliterative phlebitis (Figure 7), characteristic histopathological features of IgG4-related disease. The patient's serum IgG4 was elevated at 227 mg/dL (range 2.4–121 mg/dL). He was referred to Rheumatology for further management and has completed a course of corticosteroids with resolution of his IgG4 level to 86.6 mg/dL.

## 3. Discussion

IgG4-related disease is a newly recognized disorder that has been reported in nearly every organ system. IgG4 is the least abundant subclass of IgG, accounting for less than 5% of total IgG. It does not activate the complement pathway and therefore is thought to play little role in immune activation. IgG4 usually only responds to prolonged and repeat antigen exposure and is involved in the anti-inflammatory process. It is theorized that molecular mimicry in genetically predisposed individuals and autoimmunity are responsible for IgG4-related disease. The activation of regulatory T cells is thought to be unique to IgG4-related disease, resulting in overexpression of transforming growth factor beta and promotion of fibrosis. Patients typically have a subacute presentation, either with solitary organ involvement with silent systemic involvement or with multiorgan involvement [1].

In this patient presentation, physical examination and image findings were suspicious for metastatic adenocarcinoma of the urachus. IgG4-positive plasma cells can infiltrate tissue involved by cancer. Therefore, proper tissue sampling and pathologic review must be performed if malignancy is suspected, as the presence of IgG4-positive plasma cells in tissue is not in itself diagnostic of IgG4-related disease. An international symposium was held in Boston in 2011 to better define the histopathology of IgG4-related disease and provide guidelines for pathologic diagnosis, which is based primarily on morphologic appearance [9]. Dense lymphoplasmacytic infiltrate, storiform pattern of fibrosis, and obliterative phlebitis are the described histopathologic features of IgG4-related disease. An accurate diagnosis requires the presence of at least two of the three described features of which our patient demonstrated all three. Elevated tissue IgG4 counts and IgG4 : IgG ratios should be determined but are of secondary importance. Serum IgG4 counts are typically elevated as in our patient but have been reported normal in up to 40% of biopsy proven IgG4-related disease [10].

IgG4-related disease has been described in a wide variety of organs, but to our knowledge, this is the first reported case of IgG4-related disease presenting as a urachal tumor. This case meets the criteria for IgG4-related disease in a new organ, as outlined by the consensus statement on IgG4-related diseases [9]. Histopathological analysis demonstrated dense lymphoplasmacytic infiltration with IgG4-positive cells, storiform pattern of fibrosis, obliterative phlebitis, elevated serum IgG4, and involvement of other organ systems consisting of lymphatic and pulmonary lesions demonstrating increased metabolism on PET scan, as has been reported with IgG4-related disease [11].

Treatment of IgG4-related disease typically consists of glucocorticoid therapy but can vary based more on which organs are involved and the degree of fibrosis present. Progressive fibrosis can obviously impair organ function if left untreated and can be so extensive that it becomes unresponsive to steroid therapy. No clinical trials have been performed to determine the optimal treatment course, but other classes of immunosuppressants have been utilized with some success [1]. In our patient, surgical resection was diagnostic and therapeutic for his bladder symptoms. He has completed a course of glucocorticoids with a good serum IgG4 response.

Clearly, a high index of suspicion by pathologists and clinicians is necessary to appropriately diagnose and treat IgG4-related disease, an increasingly recognized condition. Increased awareness will hopefully improve diagnostic and treatment strategies.

## References

[1] Stone JH, Zen Y, Deshpande V (2012). Mechanisms of disease: IgG4-related disease. *The New England Journal of Medicine*.

[2] Hamano H, Kawa S, Horiuchi A (2001). High serum IgG4 concentrations in patients with sclerosing pancreatitis. *The New England Journal of Medicine*.

[3] Kamisawa T, Funata N, Hayashi Y (2003). A new clinicopathological entity of IgG4-related autoimmune disease. *Journal of Gastroenterology*.

[4] Moriarty MA, Dahmoush L, Nepple KG (2014). IgG4 related disease of the ureter (inflammatory pseudotumor). *The Journal of Urology*.

[5] Guma M, Firestein GS (2012). IgG4-related diseases. *Best Practice and Research: Clinical Rheumatology*.

[6] Buijs J, Maillette de Buy Wenniger L, van Leenders G (2014). Immunoglobulin G4-related prostatitis: a case-control study focusing on clinical and pathologic characteristics. *Urology*.

[7] Migita K, Miyashita T, Mizuno A (2014). IgG4-related epididymo-orchitis associated with bladder cancer: possible involvement of BAFF/BAFF-R interaction in IgG4-related urogenital disease. *Modern Rheumatology*.

[8] Park S, Ro JY, Lee DH, Choi SY, Koo H (2013). Immunoglobulin G4-associated inflammatory pseudotumor of urinary bladder: a case report. *Annals of Diagnostic Pathology*.

[9] Deshpande V, Zen Y, Chan JK (2012). Consensus statement on the pathology of IgG4-related disease. *Modern Pathology*.

[10] Sah RP, Chari ST (2011). Serologic issues in IgG4-related systemic disease and autoimmune pancreatitis. *Current Opinion in Rheumatology*.

[11] Horger M, Lamprecht H, Bares R (2012). Systemic IgG4-related sclerosing disease: spectrum of imaging findings and differential diagnosis. *American Journal of Roentgenology*.

